# Semi‐supervised auto‐segmentation method for pelvic organ‐at‐risk in magnetic resonance images based on deep‐learning

**DOI:** 10.1002/acm2.14296

**Published:** 2024-02-22

**Authors:** Xianan Li, Lecheng Jia, Fengyu Lin, Fan Chai, Tao Liu, Wei Zhang, Ziquan Wei, Weiqi Xiong, Hua Li, Min Zhang, Yi Wang

**Affiliations:** ^1^ Department of Radiation Oncology Peking University People's Hospital Beijing China; ^2^ Radiotherapy laboratory Shenzhen United Imaging Research Institute of Innovative Medical Equipment Shenzhen China; ^3^ Zhejiang Engineering Research Center for Innovation and Application of Intelligent Radiotherapy Technology Wenzhou China; ^4^ Department of Radiology Peking University People's Hospital Beijing China; ^5^ Radiotherapy Business Unit Shanghai United Imaging Healthcare Co., Ltd. Shanghai China

**Keywords:** auto‐segmentation, deep‐learning, semi‐supervised learning

## Abstract

**Background and purpose:**

In radiotherapy, magnetic resonance (MR) imaging has higher contrast for soft tissues compared to computed tomography (CT) scanning and does not emit radiation. However, manual annotation of the deep learning‐based automatic organ‐at‐risk (OAR) delineation algorithms is expensive, making the collection of large‐high‐quality annotated datasets a challenge. Therefore, we proposed the low‐cost semi‐supervised OAR segmentation method using small pelvic MR image annotations.

**Methods:**

We trained a deep learning‐based segmentation model using 116 sets of MR images from 116 patients. The bladder, femoral heads, rectum, and small intestine were selected as OAR regions. To generate the training set, we utilized a semi‐supervised method and ensemble learning techniques. Additionally, we employed a post‐processing algorithm to correct the self‐annotation data. Both 2D and 3D auto‐segmentation networks were evaluated for their performance. Furthermore, we evaluated the performance of semi‐supervised method for 50 labeled data and only 10 labeled data.

**Results:**

The Dice similarity coefficient (DSC) of the bladder, femoral heads, rectum and small intestine between segmentation results and reference masks is 0.954, 0.984, 0.908, 0.852 only using self‐annotation and post‐processing methods of 2D segmentation model. The DSC of corresponding OARs is 0.871, 0.975, 0.975, 0.783, 0.724 using 3D segmentation network, 0.896, 0.984, 0.890, 0.828 using 2D segmentation network and common supervised method.

**Conclusion:**

The outcomes of our study demonstrate that it is possible to train a multi‐OAR segmentation model using small annotation samples and additional unlabeled data. To effectively annotate the dataset, ensemble learning and post‐processing methods were employed. Additionally, when dealing with anisotropy and limited sample sizes, the 2D model outperformed the 3D model in terms of performance.

## 1 INTRODUCTION

In recent years, there has been a great deal of interest in developing computed tomography (CT) based auto‐segmentation methods for organ‐at‐risk (OAR) regions in radiotherapy.^1^, ^2^, ^3^ Not only does this approach significantly reduce the time required for contour delineation and review, but it also improves work efficiency.^4^ However, current auto‐segmentation models still rely heavily on large, high‐quality image collections and annotation data.^5^, ^6^, ^7^ The high cost of medical image annotation remains a significant limitation to the practical application of these models in clinical practice.

Compared to CT, magnetic resonance (MR) images offer higher soft tissue resolution and clearer boundaries for certain organs and targets^8^ that may not be discernible in CT scans. In clinical practice, MR images are utilized for daily adaptive radiotherapy targets and OAR delineation. However, the process of accurately delineating OARs from MR data can be time‐consuming. Unfortunately, only a limited number of open‐access annotation datasets are available for training segmentation models using MR images, with relatively small dataset sizes.^9^, ^10^

Auto‐segmentation in MR images using the deep‐learning method has evolved along with the methods in CT. In recent years, auto‐segmentation methods have improved with the development of Convolutional Neural Networks (CNNs) and the use of Fully Convolution Networks (FCNs).^11^ The CNN‐based segmentation network, U‐Net^12^ has significantly improved performance over edge detection algorithms. It has been proven to be suitable for medical images. Compared with 2D models, 3D models^13^ could combine spatial context information with image texture, which is more suitable for medical images. The 3D models usually have better performance than 2D models in segmentation,^14^ image translation,^15^ and dose prediction.^16^ For auto‐segmentation, residual block,^17^ bottleneck block,^18^ dense block,^19^ and attention module^20^ are used for modified U‐Net to improve segmentation accuracy. However, they are all based on supervised methods using fully annotated data to train models.

During radiotherapy, the process of annotation can be time‐consuming for both CT and MR images. Therefore, it is common to annotate OARs in CT images related to the treatment plan (CT‐sim), rather than directly annotating in MR images. This lack of MR segmentation datasets can make it challenging to develop new models that achieve state‐of‐the‐art performance on open‐access datasets.^9^, ^10^ Additionally, when these new models are applied to clinical data acquired from hospitals, they may not perform as well as expected due to variations in patient anatomy and treatment plans.

Compared to supervised deep learning methods that require high‐quality fully annotated data, the semi‐supervised methods^21^ only require small annotation samples to train a model with high performance. They have been proven useful in real‐world 2D image semantic segmentation tasks, such as mean teacher models and interpolation consistency training. However, they are complex and challenging to train. For medical image auto‐segmentation, there are some recent reports^22^ for a semi‐supervised learning method focused on reducing training sample size. In this paper, we propose a low‐cost auto‐segmentation method using semi‐supervised deep learning with small annotation samples for pelvic OARs segmentation in MR images. This demonstrates the potential for clinical applications by training a segmentation model using small annotation samples.

## 2 METHOD

### 2.1 Data set

The study consisted of 116 patients diagnosed with rectal cancer from April 2018 to March 2022 at Peking University People's Hospital. The age distribution of patients was 43−83 years old. MR of the pelvis was performed using a GE Discovery MR750 3.0T MRI scanner. The scanning sequence and parameters are as follows: High‐resolution non‐fat‐suppressed fast recovery fast spin‐echo (FRFSE) T2‐weighted imaging (T2WI) sequence: TR 3755 ms, TE 90.9 ms, slice thickness 6 mm, slice interval 7.5 mm, spacing 0.6641 and 0.6641 mm, field of view 16 cm×16 cm. Each patient also had a plan CT, scanned by Philips Brilliance Big Bore, slice thickness 3 mm, slice interval 3 mm, spacing 1.365 and 1.365 mm, field of view 70 cm × 70 cm.

**FIGURE 1:**
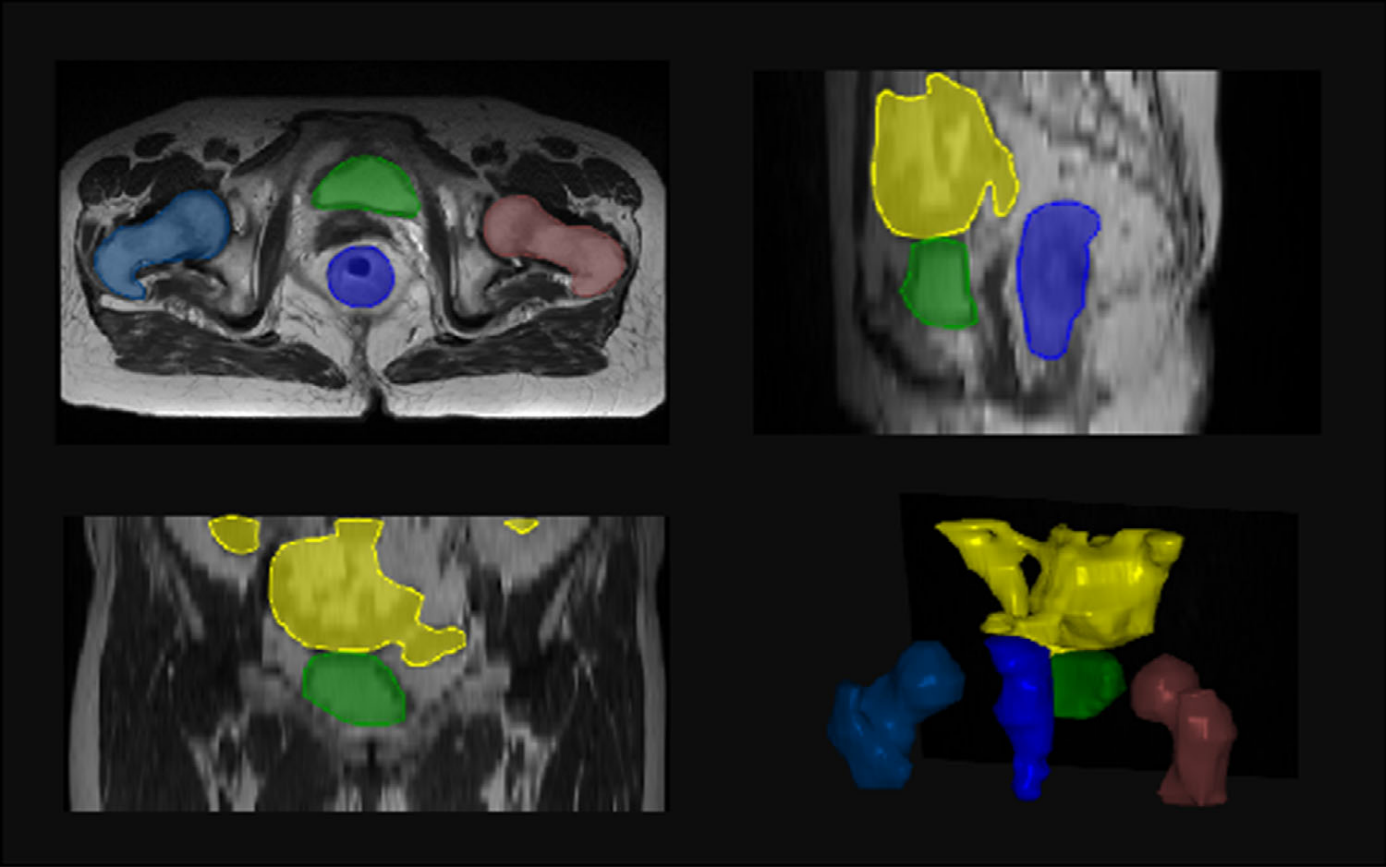
The OAR contours in MR image. Yellow: small intestine, green: bladder, blue: rectum, indigo: femoral head right and brown: femoral head left.

**FIGURE 2:**
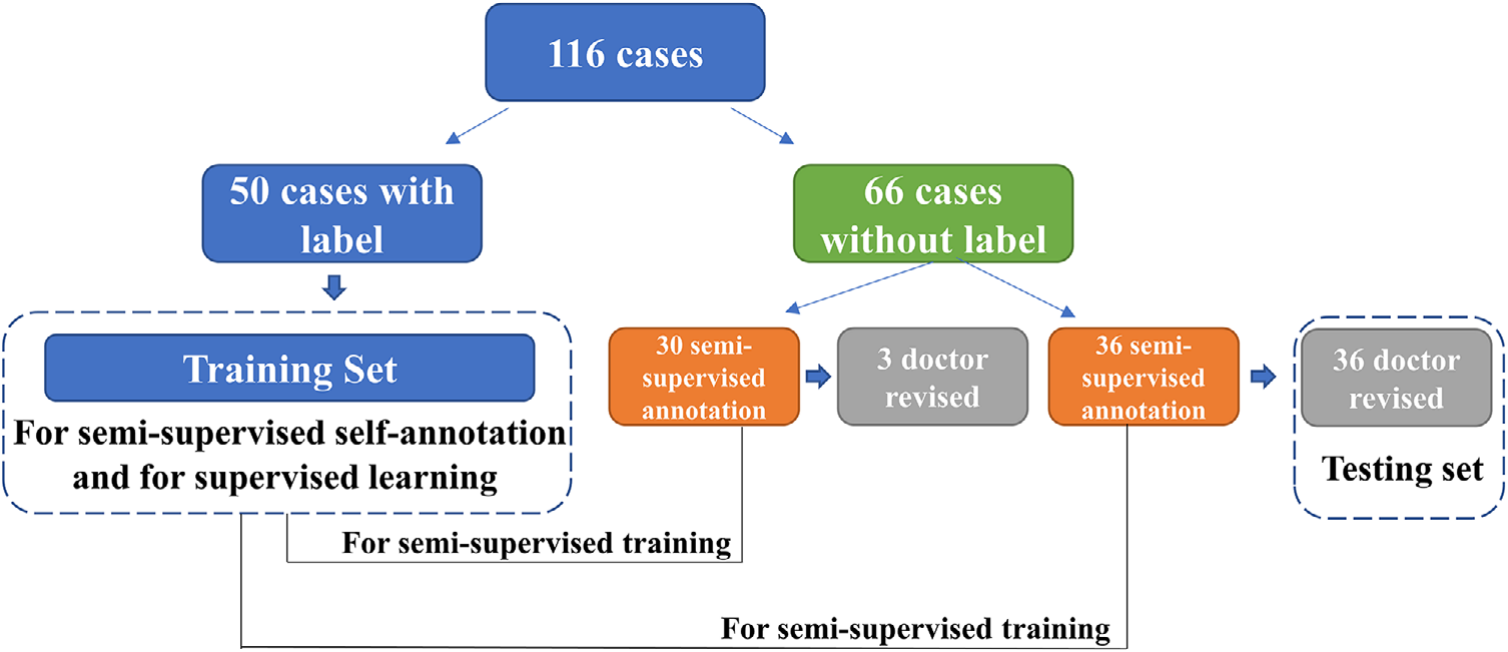
Datasets used in this manuscript. Fifty cases with labels were used for semi‐supervised self‐annotation and supervised learning training. The other cases without labels were used for semi‐supervised annotation and then revised by the doctor.

All the MR images were labeled with bladder, femoral heads, rectum, and small intestine. Two doctors revised the all‐OAR labels, as shown in Figure 1. A training set of 50 annotation samples was created. For unlabeled images, thirty cases were set as a semi‐supervised annotation training set, while the remaining images served as the test set. After self‐annotation, the doctors revised the all‐OAR labels based on the semi‐supervised annotation data. The test set refined by the doctors was used to evaluate the performance of both supervised and semi‐supervised methods, including 2D and 3D networks. The details can be seen in Figure 2.

### 2.2 Network

**FIGURE 3:**
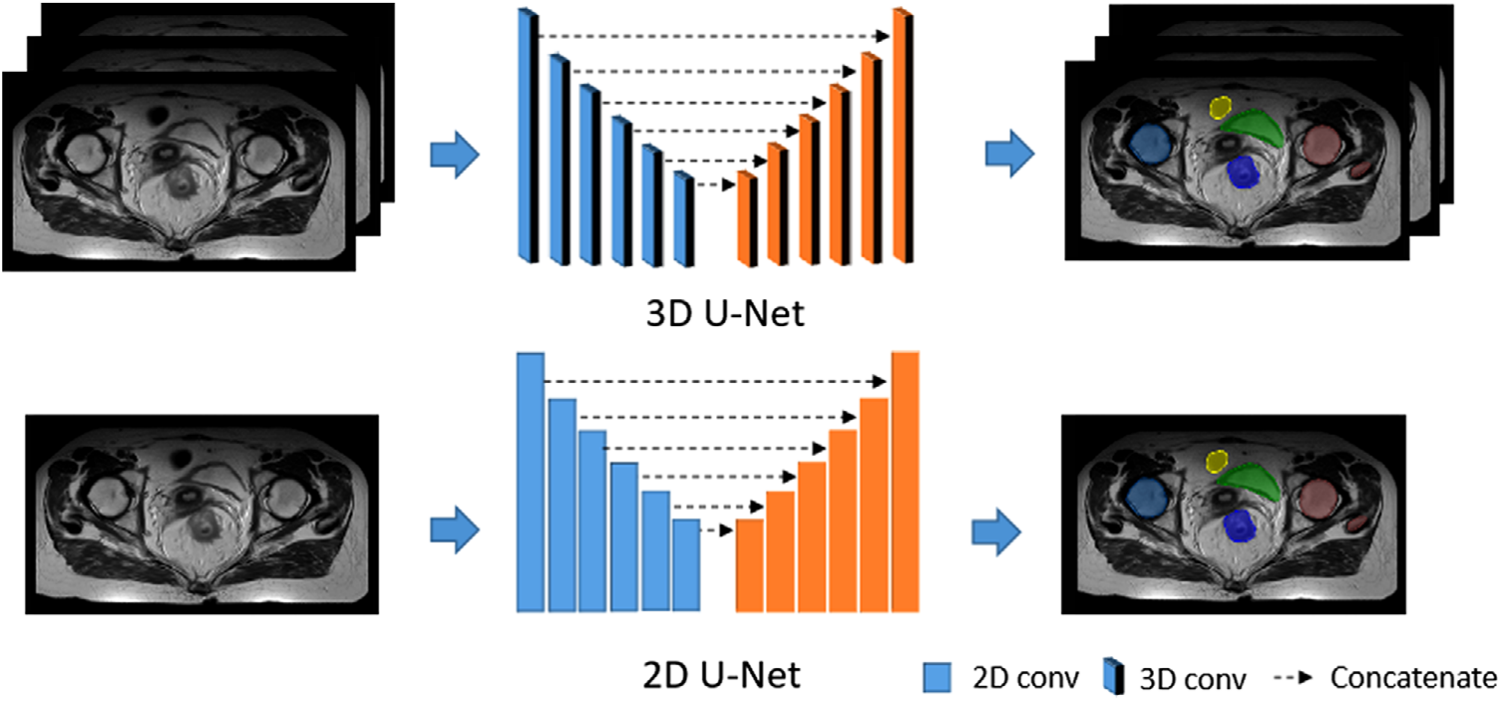
The architectures of the segmentation network: 3D U‐Net and 2D U‐Net.

In this paper, we evaluated the performance of two segmentation networks, 2D U‐Net^12^ and 3D U‐Net.^14^ Although the MR images were 3D slices, the slice thickness is anisotropic compared with image spacing. A 2D segmentation network might perform better in this situation. The architectures of the segmentation networks are shown in Figure 3. For both auto‐segmentation networks, we used convolutions, instance normalization, and leaky ReLU^23^ for each computational block. Strided convolutions and transposed convolutions were used for down‐sampling and up‐sampling, respectively. As for 3D U‐Net, the convolution kernel was [3, 3, 3], and down‐sampling was 2 along *z* and 6 along the *x* and *y*‐axis. The input patch size was [32, 256, 256] and the output was [5, 256, 256]. The convolution kernel was [3, 3] for 2D U‐Net and down‐sampling was 6 along *x* and *y* axis. For 2D U‐Net, the input patch size was [256, 256] and the output was [5, 256, 256] too. One channel was the output of the background and the other channel was the rectum, bladder, femoral heads, and small intestine respectively. To evaluate the segmentation performance, we compared the performance of 2D and 3D models in each comparative experiment.

### 2.3 Model training

For each MR slice, we used z‐score intensity normalization per image, which means we used means subtraction and division by standard deviation. Random cropping and random rotating were used for data augmentation^24^ during the training stage. The bladder, femoral heads, rectum, and small intestine were contoured as index 1, 2, 3, and 4, respectively, without overlap. The composite loss function consisting of Dice and cross‐entropy, as shown in Equation 1, which accomplished convergence fast and steadily:

(1) $$l_{loss}=a\cdot l_{dice}+\left(1-a\right)l_{cross-entropy},$$

(2) $$l_{dice}=\frac{2\left|A\cap B\right|}{\left|A\right|+\left|B\right|},$$

(3) $$l_{cross-entropy}=-\frac{1}{n}\sum_{x}y\ln p+(1-y)\ln(1-p)].$$

where *a* in Equation (1) was the weight of Dice loss in loss function in Equation (1). *A* and *B* in Equation (2) represent the prediction and reference labels, respectively. In Equation (3), *n* was the number of samples, *y* and *p* were reference labels and predictions after one‐hot encoding, and *x* was the dimension of prediction vector.

The network was built by Pytorch (version 1.6) in Python 3.7. The Compute Unified Device Architecture (CUDA) version was 10.2. And the program was run on Ubuntu 1804. The training parameters are as follows, the total epoch was set to 250, the original learning rate was 0.01, and the optimizer was Adam. The models were trained on Nvidia RTX 3090 with 24GB graphic memory. For 3D U‐Net, the patch size was [32, 256, 256], and the batch size was 2, and all data were resampled into [6.5, 0.664, 0.664]. For 2D U‐Net, the patch size was [512, 512], the batch size was 12, and all data were resampled into [0.664, 0.664]. Each epoch was trained for 88s (3D model) and 61s (2D model) in RTX 3090.

### 2.4 Semi‐supervised method for data annotation

**FIGURE 4:**
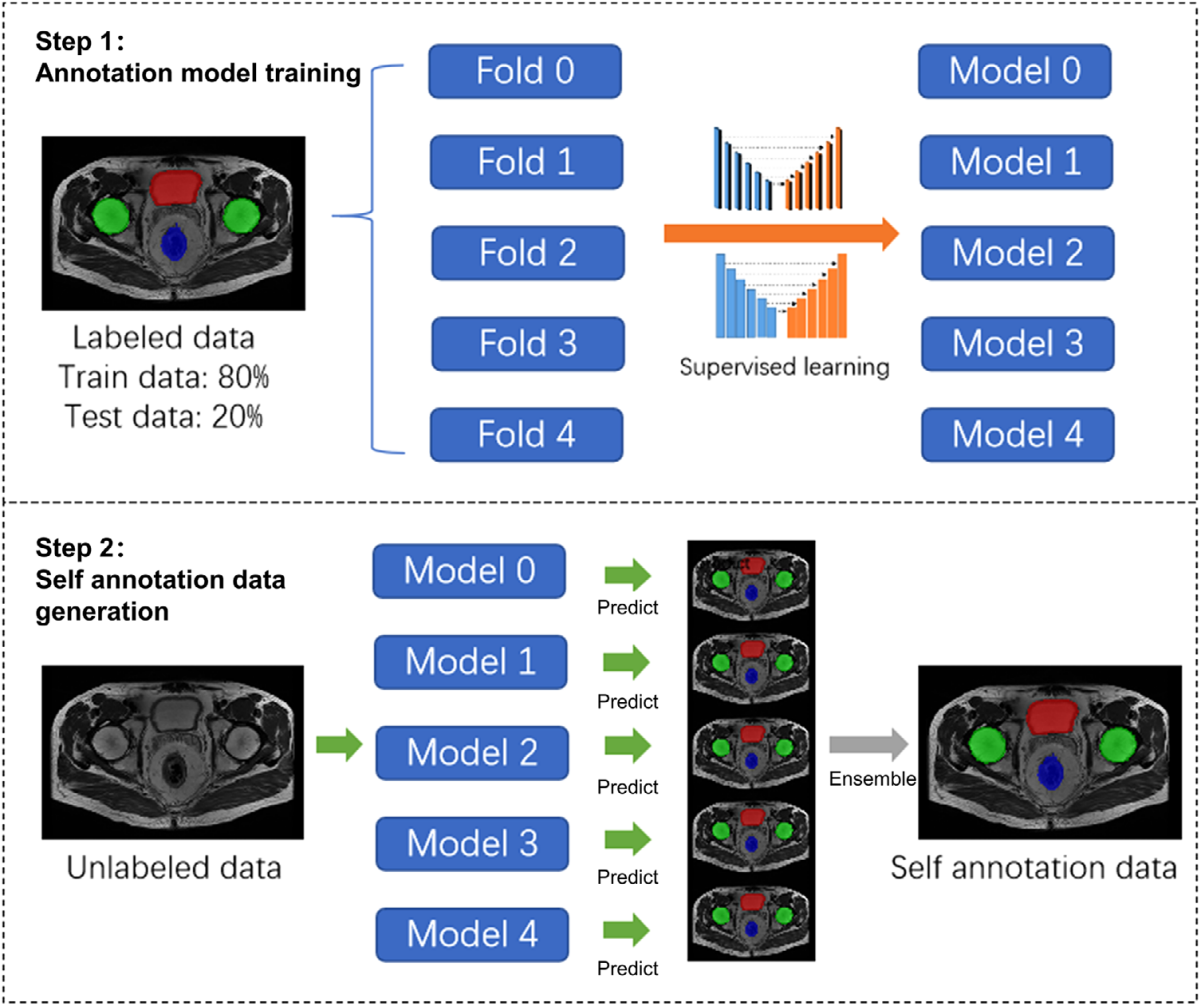
Semi‐supervised learning process including two steps. Step 1: annotation model training. Step 2: Self annotation data generation.

**FIGURE 5:**
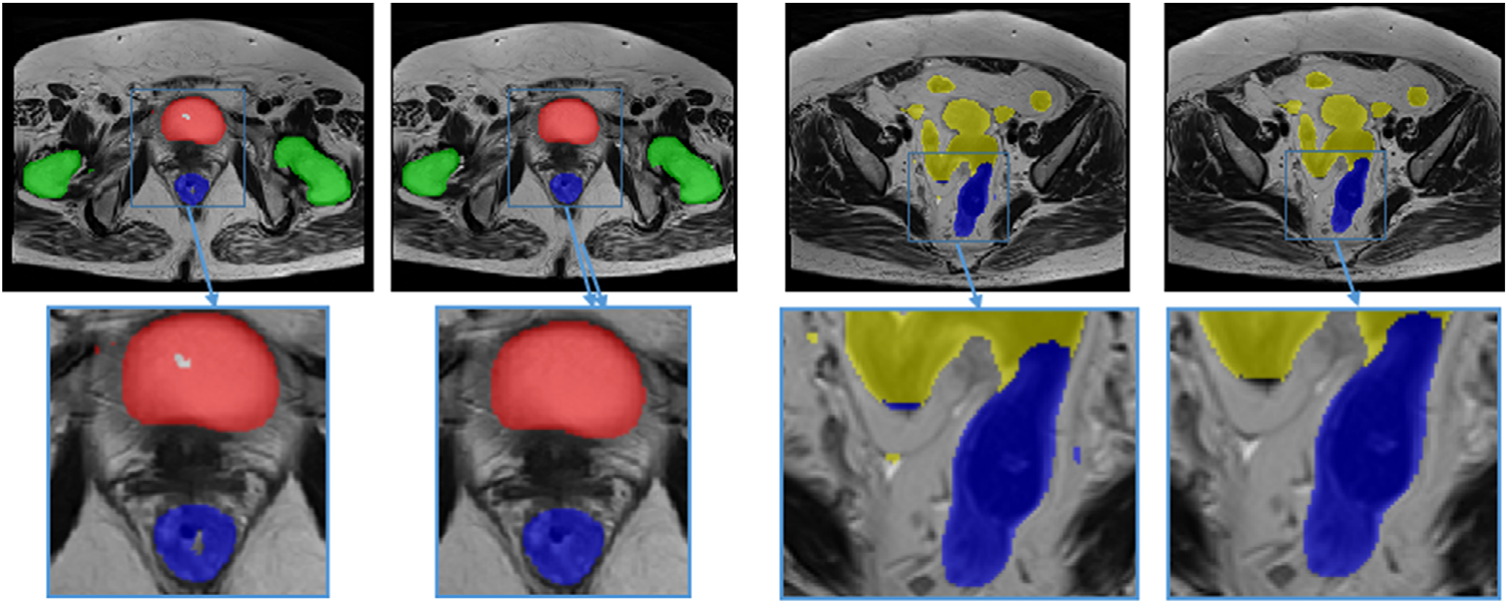
Self annotation data with post‐processing algorithms. Red: bladder, green: femoral heads, blue: rectum. After post‐processing, some mistakes like cavities, and small fragments were removed.

The semi‐supervised learning process is shown in Figure 4: The labeled data was set as the training set and the testing set. All labeled data was set into 5 folds for cross‐validation, and each fold was set as testing data for model training in turn. Then the five segmentation models were ensemble to infer the unlabeled data by average voting. The ensemble method^25^ was calculated the average value for the five folds results. Post‐processing algorithms,^26^ such as filling holes, the maximum threshold domain, and morphological method were applied for revising model annotation data, which is shown in Figure 5. The post‐processed self‐annotated data were added to the training dataset to re‐train an auto‐segmentation model, which made full use of unlabeled data. The post‐processing methods for the new model are the same as described above.

To find a suitable semi‐supervised segmentation method for pelvic OAR segmentation in MR images, the self‐annotation performance of a single model and ensemble learning of multiple models were compared. We compared the performance of supervised learning and semi‐supervised learning in single model and ensemble models using the same data. Meanwhile, we investigated the performance of supervised learning under different training data volumes and the performance of semi‐supervised learning under different annotated data ratios. The results would show the segmentation effects of the two methods on small samples.

### 2.5 Evaluation

The evaluation of auto‐segmentation methods included subjective evaluation and objective evaluation.

We calculated the Dice similarity coefficient (DSC),^27^ 95% Hausdorff distance (HD95),^28^ and average surface distance (ASD)^29^ between the results predicted by model (P) and reference label (G) for subjective evaluation. The DSC measures the relative volumetric overlap between masks, a higher value means a higher overlap ratio. A Dice value of 0 indicates no overlap between masks, while a value of 1 indicates complete overlap,

(4) $$\mathrm{DSC}=\frac{2\left|P\cap G\right|}{\left|P\right|+\left|G\right|}.$$

The HD95 represents the 95th percentile of the distances between boundary points in model‐predicted and reference masks. The HD95 reflects the agreement between two contours, a higher value indicates a larger difference,

(5) $$\begin{array}{ccc} HD(P,G) & = & \max\left\{\max_{p\in P}\left\{\min_{g\in G}\left\|p-g\right\|\right\},\right.\\ & & \left.\max_{g\in G}\left\{\min_{p\in P}\left\|g-p\right\|\right\}\right\}. \end{array}$$

The ASD^30^ counts the average distance between the surfaces of two contours,

(6) $$\begin{array}{ccc} ASD\;\left(P,G\right) & = & \frac{1}{\left|S\left(P\right)\right|+\left|S\left(G\right)\right|}\;\left(\sum_{p\in S\left(P\right)}\min_{g\in S\left(G\right)}\left\|p-g\right\|\right.\\ & & \left.+\sum_{g\in S\left(G\right)}\min_{p\in S\left(P\right)}\left\|g-p\right\|\right). \end{array}$$

where S(P) and S(G) denote the point set of prediction pixels and reference pixels respectively. The most consistent segmentation result can be obtained when ASD equals 0.

However, the evaluation indexes such as DSC, do not provide insight into how much the contours would need to be edited in clinical practice. To assess the clinical efficiency of our method, human experts were requested to further evaluate the applicability of auto‐segmentation contours. Accuracy was classified as follows^31^: no revision required, revisions required for more than 0% to 20% of the volumetric contours, revisions required for more than 20% to 40% of the volumetric contours, revisions required for more than 40% to 60% of the volumetric contours, revisions required for more than 60% to 80% of the volumetric contours, and revisions required for more than 80% to 100% of the volumetric contours. The scores were five to zero respectively.

## 3 RESULT

### 3.1 Performance of supervised auto‐segmentation models

**FIGURE 6:**
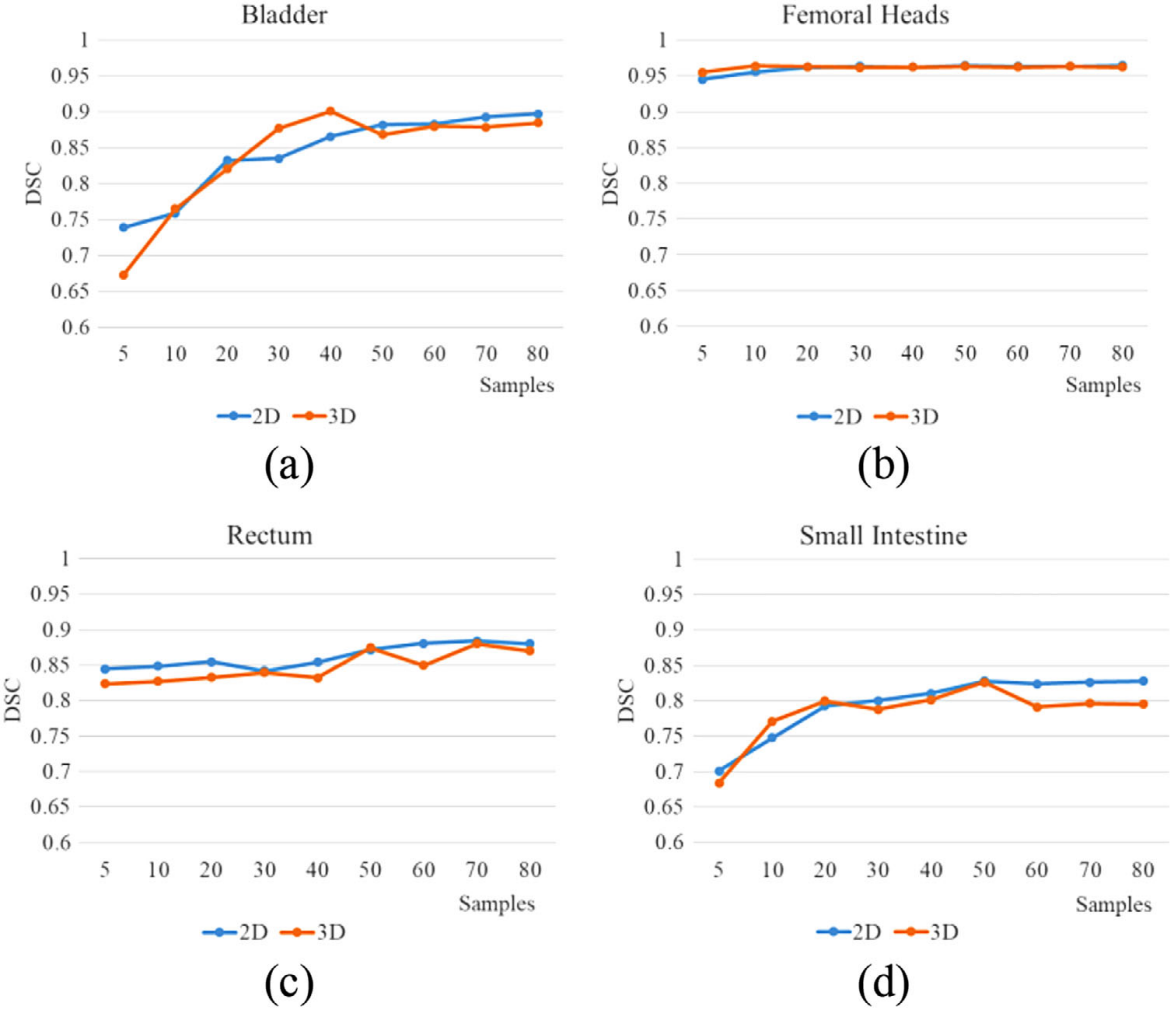
The DSC of 3D U‐Net and 2D U‐Net for the different sample sizes of the training set, (a) bladder, (b) femoral heads, (c) rectum, (d) small intestine.

The results of segmentation performance for different sample sizes of training sets between 3D U‐Net and 2D U‐Net are shown in Figure 6. As the number of samples increased in the training set, the DSC of the bladder, rectum, and small intestine increased synchronously. The DSC of the femoral head was stable. 2D models show better performance, especially for small intestine.

### 3.2 Performance of semi‐supervised auto‐segmentation models

**FIGURE 7:**
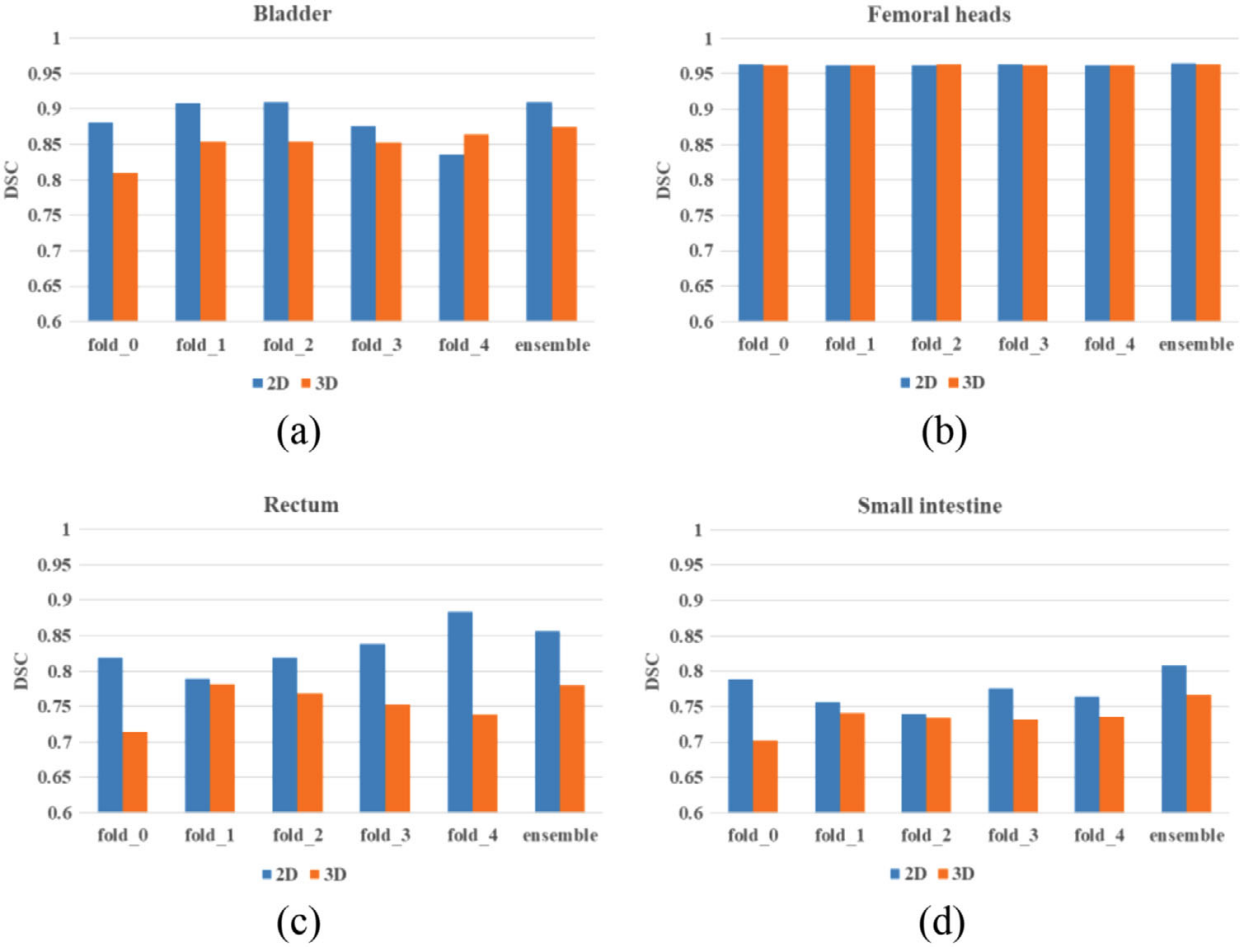
The DSC of 3D U‐Net and 2D U‐Net of five different OARs with semi‐supervised learning method, (a) bladder, (b) femoral heads, (c) rectum, (d) small intestine.

The results of segmentation performance in small sample size labeled data between 3D U‐Net and 2D U‐Net are shown in Figure 7. The DSC of each OAR using different validation folds was evaluated. Compared with the single model, the ensemble method showed better performance, while it needs more training time and sub‐models. Although 3D models had more parameters and spatial information, in this research, 2D models were better than 3D models.

To compare the performance of our method and the single fully supervised model method, we trained 2D U‐Net (nnUNet) by the same datasets revised by doctors on behalf of the supervised method, respectively. The performance between the semi‐supervised method and the supervised method of the 2D segmentation model is shown in Tables 1 and 2. The semi‐supervised method shows better performance than supervised method. We also evaluated the performance of the semi‐supervised method for 50 labeled data and only 10 labeled data. The result was surprising and shown in Table 3. Using only 10 labeled samples and the other 70 unlabeled samples, the performance was not decreased significantly compared to those using 50 unlabeled samples. The semi‐supervised method can achieve the performance of the supervised method using small labeled samples.

**TABLE 1:** The performance of the semi‐supervised method and the supervised method of ensemble learning.

	Semi‐supervised method				Supervised method			
OARs	DSC	HD/mm	HD95/mm	ASD/mm	DSC	HD/mm	HD95/mm	ASD/mm
Bladder	0.964	3.120	0.605	0.017	0.909	16.576	1.215	0.296
Femoral heads	0.999	0.083	0	0.001	0.982	5.917	0.563	0.061
Rectum	0.918	42.447	7.740	1.612	0.882	85.207	4.632	0.958
Small intestine	0.880	56.329	16.462	0.168	0.844	53.859	9.369	1.803

**TABLE 2:** The performance of the semi‐supervised method and the supervised method of single model.

	Semi‐supervised method				Supervised method			
OARs	DSC	HD/mm	HD95/mm	ASD/mm	DSC	HD/mm	HD95/mm	ASD/mm
Bladder	0.954	6.973	0.595	0.078	0.896	12.842	1.718	0.425
Femoral heads	0.984	4.768	0.624	0.053	0.984	4.4688	0.488	0.053
Rectum	0.908	54.436	5.238	0.975	0.890	71.296	4.011	0.813
Small intestine	0.852	83.792	18.046	1.290	0.828	45.229	9.743	1.706

**TABLE 3:** The performance of the semi‐supervised method with different labeled sample sizes.

	Semi‐supervised method/50 labeled samples				Semi‐supervised method/10 labeled samples			
OARs	DSC	HD/mm	HD95/mm	ASD/mm	DSC	HD/mm	HD95/mm	ASD/mm
Bladder	0.954	6.973	0.595	0.078	0.944	7.722	0.730	0.118
Femoral heads	0.984	4.768	0.624	0.053	0.982	4.743	0.651	0.061
Rectum	0.908	54.436	5.238	0.975	0.896	52.117	6.191	1.146
Small intestine	0.852	83.792	18.046	1.290	0.842	87.182	20.052	1.411

### 3.3 Assessment of auto‐segmentation models by human experts

**FIGURE 8:**
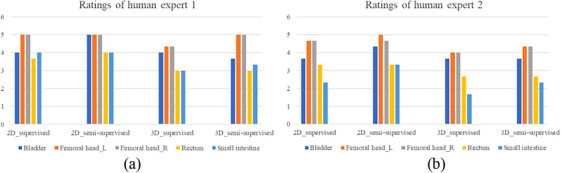
Accuracy scores of different models by two human experts.

For objective evaluation, two human experts modified the results predicted by models and gave them a rating. The accuracy of the 2D and 3D segmentation models of supervised and semi‐supervised using the single model is shown in Figure 8. Femoral heads required little revision, and the semi‐supervised method could improve the accuracy of auto‐segmentation models. The 2D models show better performance than the 3D models.

### 3.4 Inference time and parameter quantity

**FIGURE 9:**
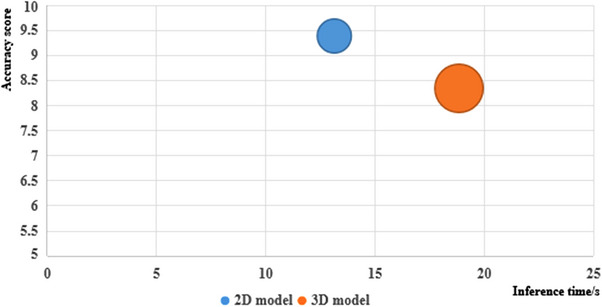
The accuracy score, inference time, and parameters of 2D and 3D model.

The inference time and parameters of the 2D and 3D models with the semi‐supervised learning method were shown in Figure 9. The accuracy score was the sum of each OAR of DSC and one‐fifth of average human experts’ scores. For the 2D model, the accuracy score was 9.41, the inference time was 12.79 s for one case, and the parameter quantity was 29.97 million. For the 3D model, the accuracy score was 8.33, the inference time was 18.30 s, and the parameter quantity was 44.80 million. The 2D model outperformed 3D model with a higher accuracy score, shorter inference time, and fewer parameters.

## 4 DISCUSSION

In this paper, we proposed a semi‐supervised deep‐learning method using only small annotation data and other unlabeled data for pelvic OAR segmentation in MR images. In this manuscript, we compared the performance of supervised learning on different sizes of training sets. The results show that supervised learning required a large amount of labeled data, and in the case of less labeled data, the performance of segmentation would drop sharply. Using 10 and 80 labeled data for the 2D supervised learning method, the DSC of the bladder, femoral heads, rectum, and small intestine was 0.689, 0.956, 0.709, and 0.69 to 0.811, 0.964, 0.731, and 0.778. However, with only 10 labeled data, the DSC of the corresponding OARs was 0.944, 0.982, 0.896, and 0.842. Compared with 50 labeled data, the performance was not significantly performance degradation. In other related studies, Fu^32^ provided a deep‐learning based approach to segment five pelvic organs, including prostate, bladder, rectum, and femoral heads using 100 cases. The DSC was 0.96, 0.91, 0.93, 0.95, and 0.95. Li^33^ provided a patient‐specific deep‐learning auto‐segmentation for MR‐guided adaptive radiotherapy. The average DSC was 0.90 and the contouring time was reduced significantly (*p* < 0.05) using the proposed method (73.4 ± 6.5 s) compared to the manual process (12 ∼ 22 mins). The performance of the method we proposed was almost the same as other related studies and could meet the requirements of clinical application.

The key point of the proposed method was how to improve the accuracy of semi‐supervised annotation. We used 5‐fold cross‐validation for model ensemble and post‐processing algorithms, which are necessary during the semi‐supervised annotation stage due to the increased training time and calculation resources. The results show that it could improve accuracy and reduce obvious errors in labeling. During the clinical model inference stage, a single model can satisfy performance requirements while being lightweight and efficient.

Subjective and objective assessments have demonstrated similar conclusions. Femoral heads in MR images have clear outlines, making them easy to segment for all segmentation methods. Additionally, the accuracy of rectum and small intestine segmentation has significantly improved after using semi‐supervised annotation data for training.

We compared the performance of 2D and 3D models in each section. In this study, MR images were anisotropic with a spacing of 0.6641 mm and thickness of 6 mm. For both supervised and semi‐supervised learning methods, the 2D model shows a better performance. We also found that while the 3D models are more complex and suitable for medical data, which were also 3D data with spatial information, the 2D models showed better performance, less training time, and fewer parameters, which is shown in Figure 9. Therefore, anisotropy and isotropy of medical images are important factors to consider when choosing a model.

Compared to other research on improving auto‐segmentation models using open‐access datasets with annotation, such as new network structures,^34^ new model modules,^14^ new training strategies,^30^ and new loss functions,^35^ which is based on the premise of using the same annotation data. Instead of modifying the structure of the model or network, training strategy, or loss function, we used a basic U‐Net based auto‐segmentation model to demonstrate that semi‐supervised annotation with the model ensemble and post‐processing can generate qualified annotation data for auto‐segmentation model training in MR images. This approach addresses the problem of obtaining high‐quality annotation data for medical image segmentation to a certain extent. For semi‐supervised methods, researchers try to use limited annotations for medical image segmentation, including adding a trust module to re‐evaluate the pseudo labels^36^ from the model outputs, generating pseudo labels through transferring semantics,^37^ and proposing neighbor matching to generate pseudo‐labels on a weight basis^38^ according to the embedding similarity with neighboring labeled data. Compared with other semi‐supervised methods, the proposed method is easy to train the model and has lower training costs.

In radiotherapy, OARs and targets are typically defined in CT images. Research on auto‐segmentation algorithms is more based on CT. Therefore, collecting annotation data for MR images can be challenging. This is due to the supervised deep learning method used in clinical applications, which requires a large amount of annotated data. In particular, for pelvic MR images, manual labeling by doctors is time‐consuming and laborious. Therefore, the proposed method is beneficial for training auto‐segmentation models using other unlabeled data in clinical settings, such as MR images from other regions, PET/CT scans, etc.

There are also some limitations in our study. One of the limitations of the proposed study was that we didn't make comparison with other self‐supervised and semi‐supervised learning methods. And the data we used in this study were all pelvic data. The threshold of the data for training a robust semi‐supervised model was difficult to determine. When there is less available labeled data, semi‐supervised learning can be attempted. What's more, in this manuscript, the 2D models were more suitable than the 3D models for anisotropic images. However, we need to collect more similar data to verify this conclusion.

In the future, we will delve deeper into the potential applications of semi‐supervised learning in medical image segmentation. This approach can save radiologists time by eliminating the need to repeatedly delineate contours and minimize manual data review and modification. Additionally, we can further harness the impressive learning capabilities of deep learning techniques to enhance semi‐supervised learning algorithms and explore the representational abilities of smaller sample sizes.

## AUTHOR CONTRIBUTIONS

Xianan Li and Lecheng Jia contributed to the conception and design of the study. Yi Wang and Min Zhang provided guidance. Fan Chai and Tao Liu completed the data collection. Lecheng Jia and Fengyu Lin designed the deep‐learning model and trained the model. Weiqi Xiong drafted the manuscript, and Ziquan Wei made revisions to the manuscript. Hua Li and Xianan Li evaluated the performance of auto‐segmentation method. Yi Wang supervises and manages the project. All authors contributed to the article and approved the submitted version.

## CONFLICT OF INTEREST STATEMENT

The authors declare no conflicts of interest.
